# Oocyte polarized light microscopy, assay of specific follicular fluid metabolites, and gene expression in cumulus cells as different approaches to predict fertilization efficiency after ICSI

**DOI:** 10.1186/s12958-017-0265-2

**Published:** 2017-06-23

**Authors:** Alberto Revelli, Stefano Canosa, Loredana Bergandi, Oleksii A. Skorokhod, Valentina Biasoni, Andrea Carosso, Angela Bertagna, Milena Maule, Elisabetta Aldieri, Maria Diletta D’Eufemia, Francesca Evangelista, Nicola Colacurci, Chiara Benedetto

**Affiliations:** 1 Chair of Gynecology and Obstetrics 1, Physiopathology of Reproduction and IVF Unit, Department of Surgical Sciences, S. Anna Hospital, University of Torino, Torino, Italy; 2 Department of Oncology, University of Torino, Torino, Italy; 3 Endocrinology Laboratory, Department of Internal Medicine, University of Torino, Torino, Italy; 4 Department of Medical Sciences, Cancer Epidemiology Unit, University of Torino, Torino, Italy; 5 Department of Woman, Child, General and Special Surgery, Second University of Napoli, Naples, Italy

**Keywords:** Oocyte, Fertilization, Follicular fluid, Cumulus cells, Amh, Polarized light microscopy, Oxidative stress, AMH receptor type II

## Abstract

**Background:**

The complex relationship between oocyte morphology, specific follicular fluid metabolites, gene expression in cumulus granulosa cells, and oocyte competence toward fertilization and embryo development still needs further clarification.

**Methods:**

Forty-six oocytes retrieved from the largest pre-ovulatory follicle of patients undergoing intra-cytoplasmic sperm injection (ICSI) were considered assessing: (a) oocyte morphological characteristics at polarized light microscopy (PLM), (b) specific follicular fluid (FF) metabolites previously suggested to influence oocyte competence (AMH, markers of redox status and of cytotoxicity), (c) transcription of *AMH* and *AMH type II receptor* genes in cumulus cells. Data were analyzed using mono-parametric tests and multivariable logistic analysis in order to correlate morphological and biochemical data with fertilization.

**Results:**

Comparing normally fertilized oocytes (*n* = 29, F group) with unfertilized (*n* = 17, nF group) we observed that: (a) the meiotic spindle area and major axis were significantly higher in nF group and in fertilized oocytes undergoing an early embryo development arrest; (b) AMH level in FF was comparable in F and nF groups; (c) the FF of nF group contained significantly higher levels of cytotoxicity (lactate dehydrogenase) and oxidative stress (Cu,Zn-superoxide dismutase, catalase, 4-hydroxynonenal-protein conjugates) markers; (d) cumulus cells of nF group showed significantly higher *AMH receptor type II* gene expression.

**Conclusions:**

Taken together, these observations suggest that an excessive cytotoxicity level can alter AMH signal transduction within cumulus cells, in turn leading to partial inhibition of aromatase activity, altered cytoplasmic maturation and increased oxidative stress, factors able to impair oocyte fertilization competence and embryo growth.

**Electronic supplementary material:**

The online version of this article (doi:10.1186/s12958-017-0265-2) contains supplementary material, which is available to authorized users.

## Background

The selection of oocytes with high developmental competence is important to achieve an optimal outcome of human in vitro fertilization (IVF) avoiding ethical (and somewhere legal) controversies linked to embryo overproduction and cryostorage. Until now, oocyte selection was based on the morphological analysis of intra and extra-cytoplasmic features of the cumulus-oocyte complex (COC): cumulus cells, zona pellucida (ZP), perivitelline space and first polar body, cytoplasm and nucleus [1]. The assessment of oocyte morphology, however, has never been fully satisfactory, allowing to roughly identify negative, rather than positive, predictors of oocyte competence [2].

In the last years, new strategies of oocyte selection were tested. Among these, Polarized Light Microscopy (PLM) allows the non-invasive study of anisotropic structures of the oocyte (ZP and meiotic spindle - MS), using a circularly polarized light ray that is slowed down by well-organized, anisotropic cell structures. The birefringent signals that are generated may be measured as ″light ray retardance″ by a specific computerized image-analysis system [3]. Some studies showed that PLM is useful to assess cytoplasmic maturation, and is helpful to identify oocytes with the best potential for fertilization [4, 5] embryo growth [4, 6], blastocyst formation [7, 8] and implantation [9, 10].

As the oocyte matures surrounded by follicular fluid (FF), in intimate contact with the cumulus cells (CC), both the CC and the FF were studied to acquire indirect information on the oocyte. Among the bulk of studies on FF metabolites (reviewed in Revelli et al., 2009) [11], a few suggested that the local concentration of anti-Müllerian Hormone (FF-AMH) could indicate the fertilization potential and development capacity of the respective oocyte [12, 13]. Also the intra-follicular balance between oxidative stress and antioxidant systems, known to play a physiological role during follicle growth, was regarded as a factor able to affect oocyte competence. Unfortunately, most studies addressing the relationship between oocyte competence and FF content used pooled FF and finally reached inconsistent conclusions [14–18].

The present study was aimed at studying the relationship between oocyte competence toward fertilization and the follicle in which it matured using the assessment of: (a) oocyte morphological characteristics at PLM, (b) AMH levels, oxidative status and cytotoxicity markers in the FF, and (c) transcription of the genes for AMH and AMH type II receptor in the CC.

## Methods

### Patients

The study included 46 healthy women aged 34–42 years (mean ± SD: 36.9 ± 4.5), with normal body mass index (BMI), serum day 3 FSH < 12 IU/l, serum AMH > 1.2 ng/ml, antral follicle count (AFC) > 8, who were randomly chosen among patients undergoing intra-cytoplasmic sperm injection (ICSI) at our IVF Unit for severe male infertility.

Their clinical characteristics and the outcome of controlled ovarian stimulation (COS) were recorded, including the length of COS, the total dose of exogenous FSH, the peak circulating estradiol (E2), the number of retrieved COCs, the ovarian sensitivity index (OSI = retrieved COCs × 1000/total gonadotropin dose; Huber et al., 2013 [19]), the proportion of mature oocytes and the ratio of correctly fertilized (two-pronuclear embryos)/inseminated oocytes.

The study was carried out in accordance to the Declaration of Helsinki and was authorized as an observational study by the local Ethical Committee. A signed, written consent was obtained from all patients accepting to be included.

### Reagents

Unless otherwise specified, reagents were purchased from Sigma Aldrich (Milan, Italy), whereas plastic ware was from Falcon (Becton Dickinson, Franklin Lakes, NJ).

### Controlled ovarian stimulation and oocyte collection

COS was carried out using the gonadotropin-releasing hormone (GnRH)-agonist ″long″ protocol with recombinant FSH (Gonal-F®, Merck-Biopharma, Darmstadt, Germany) at individually tailored daily dose (100–375 IU s.c.). Follicular growth was monitored by serial measurements of circulating E2 and by transvaginal US examination performed every second day from stimulation day 7, and the dose of FSH was adjusted accordingly. When at least two follicles reached 18 mm mean diameter, with appropriate E2 levels, a single s.c. injection of 10,000 IU hCG (Gonasi HP, IBSA, Pambio Noranco, Switzerland) was administered in order to trigger ovulation. US-guided oocyte retrieval (OPU) was performed 35–37 h later under local anaesthesia (paracervical block).

The largest available follicle of each patient (overall 46 follicles of 16–23 mm diameter, mean ± SD 20.1 ± 2.4 mm) was punctured first, and its FF was immediately observed under stereomicroscope to retrieve the corresponding COC. The COC was then washed in buffered medium and individually stored; the corresponding FF was also stored for further analysis. After puncturing the largest follicle, OPU was continued with the standard procedure puncturing all other available follicles. Shortly after OPU, the oocyte contained in the largest follicle was separated from the corresponding CC by gently pipetting in a single drop of 100 μl HEPES buffered medium containing 80 IU/ml hyaluronidase (Synvitro™ Hyadase, Origio Medicult, Måløv, Denmark); it was then examined using PLM just before being injected.

### Polarized light microscopy (PLM)

**Fig. 1:**
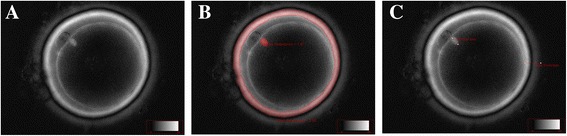
Representative images of PLM analysis of the oocyte. Automated detection by Oosight™ software of birefringent structures (in grey) of the oocyte (**a**); the ring-like structure and the ovoid region (in red) correspond to the automatic detection of the retardance, area and thickness of the inner layer of the zona pellucida (IL-ZP) and of the retardance and area of the meiotic spindle (MS) respectively (**b**); the total thickness of the ZP and the major axis length of the MS were manually measured using a line scan as depicted (**c**)

During PLM assessment, each oocyte was placed on a glass bottom dish (Willco Wells, Amsterdam, The Netherlands) in a 10 μl drop of buffered, pre-warmed medium, covered by mineral oil (Culture Oil, Cook Ltd., Ireland), and was kept on a 37 °C stage warmer under the microscope (CRi Oosight™, Woburn, MA, U.S.A.). PLM images of the oocyte were collected at 400X magnification and recorded. The Oosight Meta™ software, allowing automatic ZP and MS detection, was used to acquire and analyse data as previously described [10]. The following parameters were automatically measured: average retardance, area and thickness of the inner layer of the ZP (IL-ZP), average retardance and area of the MS (Fig. 1). In addition, the major axis of the MS and the thickness of the whole ZP were manually measured using a line scan; in detail, ZP thickness was measured at four different points and the average value was calculated as previously described by Shen, 2005 [9].

### Preparation of semen samples, ICSI, fertilization and embryo growth

Semen samples were examined to assess sperm concentration, motility, and morphology according to the World Health Organization guidelines (WHO, 2010) [20], and then were prepared by density gradient centrifugation in order to select normally motile, morphologically normal spermatozoa.

ICSI was performed on all available, mature oocytes, after 16–18 h incubation in controlled atmosphere. Normal fertilization was assessed by evaluating the presence of two pronuclei (2PN) and the extrusion of the second polar body.

The oocytes retrieved from the largest follicle of each patient were cultured individually, and divided after fertilization assessment into two groups: fertilized (F group) and non fertilized (nF group).

The zygotes were kept in single culture. Embryo morphology was evaluated first after 2 days of in vitro culture using the evidence-based, 1–10 points scale score by Holte et al., 2007 [21]; embryos with score ≥ =9 were considered top quality, whereas those with score ≤ 6 were considered of poor quality. Development to the blastocyst stage was then evaluated on day 5 according to The Istanbul Consensus Workshop (2011) [22].

### Preparation of FF samples

Forty-six FF samples (2.8–4.0 ml) derived from the largest follicle of each patient were individually collected during OPU and immediately centrifuged at 12,000 rpm for 10 min. Cellular component and debris were discarded, whereas the clear supernatant was filtered and immediately frozen at −20 °C until analysis. The protein content of the FF clear supernatant was assessed using the Bio-Rad Protein Assay (Bio-Rad, Hercules, CA, USA).

### Biochemical measurements in FF

- *FF-AMH*
  AMH concentration in 100 μl FF was measured using a second-generation enzyme-linked immunosorbent assay (ELISA) (Immunotech Beckman Coulter Laboratories, Villepinte, France), having analytical sensitivity of 0.1 ng/ml, intra- and inter-assay coefficients of variation of 4.0 and 4.6%, respectively. The FF-AMH concentration was calculated by a calibration curve and expressed as ng/ml.

b) s-E2 and *FF-E2*
  s-E2 and FF-E2 levels in 100 μl serum and 100 μl FF, respectively, were measured using a specific electro-chemiluminescence immunoassay (Cobas 117 6000 analyser fertility/hormones series, Roche Diagnostics, Indianapolis, IN, USA) having analytical sensitivity of 5.00 pg/ml. The AMH concentration was calculated by a calibration curve and expressed as pg/ml.

c) *Lactate dehydrogenase (FF-LDH) leakage*
  50 μL of FF were added to 200 μL of TRAP (82.3 mM triethanolamine, pH 7.6) supplemented with 0.5 mM sodium pyruvate and 0.25 mM NADH to start the reaction. The reaction was followed for 10 min, measuring the absorbance at 340 nm (37 °C) with a Packard EL340 micro plate reader (Bio-Tek Instruments, Winooski, VT) [23]. Each reaction kinetic was linear throughout the time of measurement. As LDH leaks out from damaged cells, extracellular LDH activity was used as an indicator of cytotoxicity of cumulus cells and it was expressed as pmol/NADH oxidized/mg of FF supernatant proteins.

d) *Cu,Zn-Superoxide dismutase (SOD) and catalase activity*
  As the levels of reactive oxygen species in FF resulted to be poorly detectable in preliminary experiments, we investigated SOD and catalase activities as enzymes representative of the local antioxidant defense.
  SOD activity was measured in 50 μl clear FF by a specific SOD assay (Trevigen, Tema Ricerca Srl, Bologna, Italy) using the xanthine/xanthine oxidase/nitroblue tetrazolium (NBT) system to generate and quantitate superoxide ion, which prevents superoxide-mediated reduction of NBT and lowers the yield of blue NBT-diformazan [24]. Briefly, 50 μl of FF clear supernatant were added to 200 μl of reaction mix containing xanthine solution, xanthine oxidase and NBT, and it was immediately assayed for total SOD and specific Cu,Zn-SOD activity using a Lambda 3 spectrophotometer (Bio-Rad Laboratories, Hercules, California) set at 560 nm. To evaluate the contribution of Cu,Zn-SOD activity to the total activity, Mn-SOD and Fe-SOD were inactivated by adding 100 μl of chloroform/ethanol (37.5/62.5, *v*/v) to the supernatant before the test [25]. Cu,Zn-SOD activity was expressed as units per mg of clear supernatant proteins.
  Catalase activity was measured in 50 μl clear FF by spectrophotometric method [26]. The reduction of H_2_O_2_ at 240 nm was recorded on UV/vis spectrophotometer at 25 C. The amount of enzyme activity that decomposed 1 mmol of H_2_O_2_ per minute was defined as one unit of activity. Specific activity was expressed as mIU/mg of FF clear supernatant proteins.

e) *4-hydroxynonenal (HNE)-protein conjugates assay*
  The accumulation of 4-HNE-protein conjugates, expressing the local oxidative status as the final product of lipo-peroxidation, was quantified by Western Blotting analysis. Proteins were extracted from 30 μl of FF clear supernatant using SDS-containing, modified Laemmli buffer (final concentrations TRIS-HCl 60 mM, EDTA 1 mM, 5% glycerol, SDS 2%, pH 6.8), supplemented with a protease inhibitor cocktail (Complete®, Roche Diagnostics S.p.A., Milano, Italy) with patent protected composition, which inhibits a broad spectrum of serine, cysteine, and metallo-proteases, as well as calpains) without β-mercaptoethanol at 95 °C for 5 min. Aliquots were kept at −20 °C prior to use and β-mercaptoethanol (5% *v*/v) was added to protein samples before loading to the SDS-PAGE. The quantified and solubilized proteins (20 μg/line) were separated with a 10% acrylamide (*w*/*v*) SDS-PAGE and transferred onto a nitrocellulose membrane (Amersham Biosciences, Fairfield, Connecticut) in order to identify 4-HNE-protein-conjugates.
  Ponceau S staining was used to verify the loading protein amount and the equality of protein pattern for each lane; the arbitrary optical density unit was acquired and compared using ImageJ software (version 1.46, Wayne Rasband, National Institutes of Health, Bethesda, MD, USA). As no differences were detected neither in loaded protein quantity, nor in total protein pattern between F and nF lines, the bovine serum albumin (BSA)-blocked membranes (saturated with 5% (*w*/*v*) BSA dissolved in phosphate-buffered saline – Tween 0.1%) were subjected overnight to the mouse monoclonal anti-4-HNE-conjugates antibody at 1:2000 dilution (clone HNEJ-2, Abcam, Cambridge, UK) at 4 °C [27]. Then the membranes were washed with PBS-Tween 0.1% and incubated with the secondary anti–mouse horseradish peroxidase–conjugated antibody (Amersham, Bucks, United Kingdom) at 1:20,000 dilution for 1 h at room temperature. The membranes were washed again with PBS-Tween 0.1% and the antibody-positive bands were visualized by enhanced chemiluminescence (ECL), acquired and quantified with Chemidoc MP (Bio-Rad Laboratories, Hercules, California), using the PDQuest software (Bio-Rad, version 7.2) according to the manufacturer’s instructions. 4-HNE and 4-HNE-modified human serum albumin (prepared as Skorokhod et al., 2014) [28] were applied for SDS-PAGE at 0.1 μg/line, processed in parallel with the samples by the same procedure in order to test the specificity of the method and for reference during quantification. For densitometry analysis, the obtained value of all positive bands in each lane was expressed as 4-HNE arbitrary units.

### Expression of AMH and AMH receptor II genes in cumulus cells

Briefly, from each studied follicle, CC were lysed using the Power SYBR Green Cells-to-CTTM kit, and genomic DNA was removed by DNase I. Total RNA of 30 ng was reversely transcribed into cDNA at 37 °C for 60 min and 95 °C for 5 min, and qRT-PCR was carried out using Power SYBR Green Cells-to-CTTM kit (Ambion; Life Technologies Italia, Monza, Italy) for the quantification of AMH (5′-CGCCTGGTGGTCCTACAC-3′ and 5′-GAACCTCAGCGAGGGTGTT-3′), AMH receptor type II (AMH-RII) (5′-TGTGTTTCTCCCAGGTAATCCG-3′ and 5′-TGTGTTTCTCCCAGGTAATCCG-3′), as well as the housekeeping gene ribosomal protein S14 (5′-AGGTGCAAGGAGCTGGGTAT-3′ and 5′-TCCAGGGGTCTTGGTCCTATT-3′), with iCycler instrument (Bio-Rad Laboratories, Hercules, California) in 20 μl of volume reaction: Four μl of cDNA and 600 nmol/L AMH, AMH-RII, or S14 primers were added to 10 μL amplification mixture (Power SYBR GreenMaster Mix, Ambion). The RT-PCR primers used were designed with NCBI/ Primer-BLAST, synthesized by Sigma (Milan, Italy). Polymerase chain reaction amplification was performed by one cycle of denaturation at 95 °C for 10 min, 45 cycles of denaturation at 95 °C for 30 s and annealing at 60 °C for 1 min.

Standard curves, with serially diluted solutions (1; 1:10; 1:10^2^; and 1:10^3^) of cDNAs obtained as a template for each gene were included in each PCR and amplified by target-specific primer sequence to quantify the PCR baseline subtracted relative fluorescence unit. The threshold cycle (Ct) reflects the cycle number at which the fluorescence generated within a reaction crosses the threshold line.

The quantification of each sample was performed comparing each PCR gene product with S14, used as reference gene to normalize the cDNA in different samples, and expressed in arbitrary units as percentage of log initial quantity, using the Bio-Rad Software Gene Expression Quantitation (Bio-Rad Laboratories), calculated using the 2_ΔΔCT method [29].

Analyzed transcripts exhibited high linearity amplification plots (*r* > 0.98) and similar PCR efficiency (92% for AMH, 93,5% for AMH-RII, 86.5% for S14), confirming that the expression of each gene could be directly compared. The specificity of PCRs was confirmed by melt curve analysis. Non-specific amplifications were never detected.

### Statistical analysis

Patients whose oocyte retrieved from the largest follicle was normally fertilized were included in F group (*n* = 29), whereas those whose studied oocyte was not fertilized were included in the nF group (*n* = 17). Data were expressed as mean ± SD of the mean. The patients’ clinical characteristics in F and nF groups, PLM-assessed parameters of their oocytes, specific substances detected in FF, and expression of genes in CC in their largest follicle were considered as pools and compared using the two-sided *t-test* for unpaired data. Correlations between these variables were assessed by Spearman rank correlation coefficient. Statistical significance level was set at *p* = 0.05.

A multivariable logistic model was fitted to data in order to estimate the joint effects of different parameters on the probability of fertilization. To avoid overfitting, a small number of variables were chosen a priori: age, LDH, FF-AMH, FF-E2, and OSI among demographic and clinical characteristics, IL-ZP area, MS area, and MS major axis among oocyte’s morphological features assessed by PLM.

## Results

### Patients

The clinical characteristics of the 46 enrolled patients and of their COS cycles are summarized in Table 1. Patients in the F group showed a significantly higher OSI (expressing ovarian responsiveness to FSH), and a significantly higher oocyte yield than patients in the nF group, despite having received a comparable dose of exogenous FSH (Table 1). The size of the punctured largest follicle ranged from 16 to 25 mm (20.9 ± 2.5) in the F group and from 14 to 23 mm (20.3 ± 2.3) for the nF group. No significant differences were observed between the two groups in term of follicle size (*p* = 0.44). All the oocytes retrieved from the largest follicles were mature and were injected by ICSI. A schematic representation of how material from individual first/largest follicle was evaluated is provided in Additional file 1: Fig. S1.

**Table 1:** Clinical characteristics of patients and of their controlled ovarian stimulation (COS) cycle according to fertilization of the oocyte retrieved from the largest follicle

	F group (*n* = 29)	nF group (*n* = 17)	*p*
Age (years)	36.9 ± 4.7	36.9 ± 4.2	ns
BMI (kg/m^2^)	23.8 ± 3.4	23.1 ± 3.0	ns
Basal (day 3) FSH (UI/I)	6.5 ± 2.2	8.6 ± 2.8	ns
AMH (ng/ml)	2.8 ± 1.1	3.1 ± 1.9	ns
AFC	16.3 ± 7.1	13.1 ± 5.7	ns
Length of COS (days)	10.5 ± 1.7	11.2 ± 1.9	ns
Total exogenous FSH (IU)	2281 ± 1188	2544 ± 1121	ns
Peak E2 (pg/ml)	1867 ± 905	1405 ± 759	ns
Retrieved oocytes	10.7 ± 4.6	5.9 ± 2.9	*p* < 0.001
OSI	6.5 ± 4.7	3.2 ± 3.0	*p* < 0.01
Mature (MII) oocytes (%)	80.2 ± 13.6	84.9 ± 16.9	ns
Fertilized (2PN) oocytes (%)	76.2 ± 16.8	45.3 ± 26.8	*p* < 0.001
Capacitated sperm (mln/ml)	0.78 ± 0.46	0.67 ± 0.31	ns

F group = patients whose oocyte was fertilized, nF group = patients whose oocyte was not. BMI = body mass index; AFC = antral follicle count; OPU = ovum pick-up; OSI = ovarian sensitivity index = number of retrieved COCs × 1000/total FSH dose; MII = metaphase II. Values are expressed as mean ± standard deviation

Considering all the retrieved oocytes, patients in the F group also had a significantly higher overall fertilization rate in spite of a comparable sperm quality (Table 1). The two patients’ groups did not significantly differ for age, BMI, basal endocrine and ultrasound markers of ovarian reserve, and for the other variables related to COS (Table 1).

### Polarized light microscopy (PLM)

Studying with PLM the oocytes derived from the largest follicle of each patient, we correlated both the oocyte ZP and MS morphological features with the clinical characteristics of the enrolled patients in the F and nF groups. We did not observe any significant correlation for ZP, whereas a significant correlation between both area and major axis of the MS with the patient age (*p* < 0.05, *r* = 0.4), the injected FSH dose (*p* < 0.05, *r* = 0.4 and *p* < 0.01, *r* = 0.5 respectively) and the OSI (*p* < 0.05, *r* = −0.4) was observed. These data suggest that an increased MS size was associated with a higher ovarian resistance to FSH and to a more advanced reproductive age. The PLM analysis of the oocyte revealed also that the area and the major axis of the MS were significantly higher in nF oocytes (*p* < 0.01 and *p* < 0.05 respectively) whereas the other PLM variables were comparable (Table 2, Fig. 1).

**Table 2:** Polarized light microscopy (PLM) assessment of the oocytes derived from the largest follicle of each patient

	F group (*n* = 29)	nF group (*n* = 17)	*p*
IL-ZP retardance (nm)	1.8 ± 0.6	1.7 ± 0.6	ns
IL-ZP area (μm^2^)	2560 ± 647	2386 ± 462	ns
IL-ZP thickness (μm)	4.4 ± 1.6	4.0 ± 1.1	ns
Total-ZP thickness (μm)	17.8 ± 1.8	18.2 ± 2.5	ns
MS retardance (nm)	1.4 ± 0.5	1.4 ± 0.2	ns
MS area (μm2)	85.6 ± 20.3	102.8 ± 16.5	*p* < 0.01
MS major axis (μm)	11.6 ± 2.1	13.4 ± 2.3	*p* < 0.05

F group = patients whose oocyte was fertilized, nF group = patients whose oocyte was not. IL-ZP = inner layer of the zona pellucida. MS = meiotic spindle. Values are expressed as mean ± standard deviation

The PLM parameters of fertilized oocytes (*n* = 29) were not related to day 2 morphological characteristics of the 29 corresponding embryos (no significant differences between top quality (*n* = 14, score ≥ 9) and poor quality (*n* = 9, score ≤ 6) embryos). The remaining embryos (*n* = 6, score 7 or 8) were classified of mean quality and not considered in our analysis.

Differently, the area and major axis of the MS were significantly lower in embryos that reached the blastocyst stage and were transferred on day 5 (*n* = 11) (Table 3), obtaining an implantation rate of 45.4% and a live birth rate of 36.4%.

**Table 3:** PLM analysis of the oocyte and biochemical measurements in FF in relation to day 2 embryo morphology and progression to blastocyst

	TQ embryos (*n* = 14)	PQ embryos (*n* = 9)	*p*	Blastocyst (*n* = 11)	Arrested (*n* = 18)	*p*
IL-ZP Retardance (nm)	1.7 ± 0.6	2.0 ± 0.7	ns	2.0 ± 0.2	2.0 ± 0.6	ns
IL-ZP Area (μm^2^)	2394 ± 652	2711 ± 632	ns	2768 ± 714	2762 ± 492	ns
IL-ZP Thickness (μm)	3.8 ± 1.5	4.8 ± 1.7	ns	5.1 ± 1.9	4.7 ± 1.2	ns
Total-ZP Thickness (μm)	17.3 ± 1.0	18.0 ± 2.0	ns	18.1 ± 2.1	18.4 ± 1.6	ns
Spindle Retardance (nm)	1.2 ± 0.5	1.6 ± 0.2	ns	1.4 ± 0.3	1.5 ± 0.3	ns
Spindle Area (μm2)	91.2 ± 30.7	85.5 ± 16.1	ns	72.2 ± 8.5	92.7 ± 21.0	*p* < 0.05
Spindle Major Axis (μm)	11.4 ± 2.7	11.4 ± 1.3	ns	9.6 ± 0.8	12.3 ± 1.4	*p* < 0.001
ffAMH (ng/ml)	1.7 ± 1.0	1.8 ± 1.1	ns	1.5 ± 0.9	2.0 ± 1.0	ns
LDH (pmol NADH/min/mg prot)	0.2 ± 0.1	0.2 ± 0.1	ns	0.2 ± 0.1	0.2 ± 0.1	ns
SOD (mlU/mg protein)	3.7 ± 0.9	3.9 ± 1.1	ns	4.2 ± 1.7	4.0 ± 0.9	ns
Catalase (mlU/mg protein)	9.5 ± 1.0	9.1 ± 1.0	ns	9.3 ± 0.3	9.4 ± 0.9	ns

Top quality (TQ) embryos = day 2 morphological score ≥ 9; poor quality (PQ) embryos = day 2 morphological score ≤ 6.; Blastocyst = embryos reaching the blastocyst stage; Arrested = embryos who arrested their development. Values are expressed as mean ± standard deviation

### Biochemical measurements in FF

**Fig. 2:**
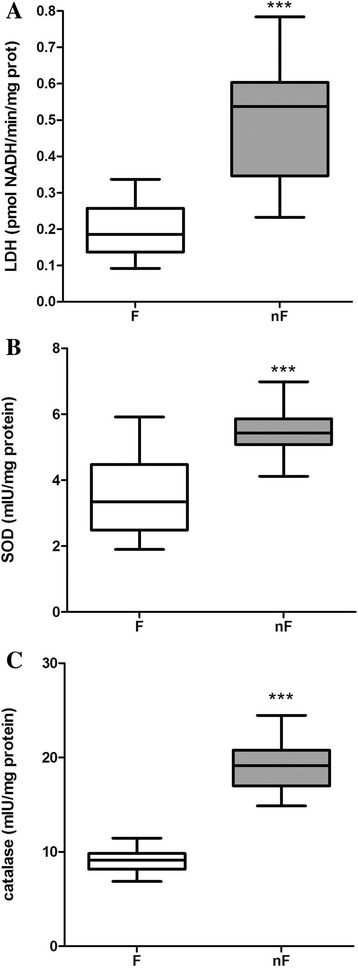
Extracellular LDH (**a**), Cu,Zn-superoxide dismutase (SOD) (**b**) and catalase (**c**) activities in the FF of the largest follicle of each patient. F group = patients whose oocyte was fertilized, nF group = patients whose oocyte was not. Measurements for each FF were performed in triplicate, and data are presented as means ± standard error of the means. F group versus corresponding nF group: *** *p* < 0.001

**Fig. 3:**
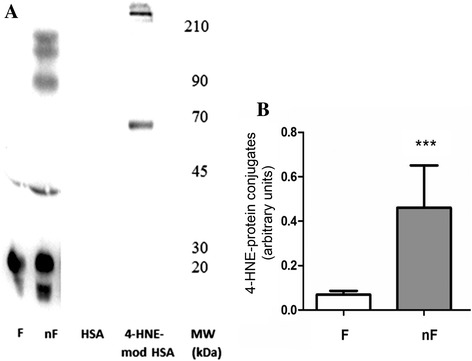
Western blotting analysis of 4-HNE-proteins conjugates in the FF. Panel **a**. Representative Western blot, obtained using antibody against anti-4-HNE-protein conjugates, of FF extracted proteins belonging to the F or nF groups. The 4-HNE-modified and non-modified non-patient human serum albumin (4-HNE-mod HSA and HSA) were used as positive and negative controls, respectively, to test the specificity of the method. The protein bands of the analyzed FFs were quantified by densitometry; the values, expressed as arbitrary units, are represented in Panel **b** as means ± standard error of the mean. F group = patients whose oocyte was fertilized (*n* = 29), nF group = patients whose oocyte was not (*n* = 17). *** *p* < 0.001

- *FF-AMH*
  Considering the largest follicle of each patient, no significant difference was observed between the F and nF groups as far as FF-AMH are concerned. FF-AMH did not correlate to embryo morphology on day 2 or to their ability to reach the blastocyst stage (Table 3). Noticeably, FF-AMH resulted significantly, directly related to serum AMH concentration (*p* < 0.05, *r* = 0.6) (data not shown).

b) *Lactate dehydrogenase (FF-LDH)*
  A significantly higher level of LDH was measured in the FFs of the nF group compared to those of the F group (*p* < 0.001; Fig. 2a). The difference between groups was remarkably high, as 75% (*n* = 13) of patients belonging to the nF group had FF-LDH ≥ 0.35 pmol NADH/min/mg proteins, whereas 65% (*n* = 19) of patients belonging to the F group had FF-LDH ≤ 0.23 pmol NADH/min/mg proteins.
  FF-LDH did not seem to affect day 2 embryo morphology or the ability to reach the blastocyst stage (Table 3).

c) *Cu,Zn-Superoxide dismutase (SOD) and catalase activity*
  The activity of Cu,Zn-SOD, that removes superoxide anions, was significantly higher in the nF group than in the F group (*p* < 0.001) (Fig. 2b). Similarly, the activity of catalase, the enzyme that catalyzes the removal of hydrogen peroxide, was significantly higher (*p* < 0.001) in nF group (Fig. 2 c). The Cu,Zn-SOD and catalase activities did not affect day 2 embryo morphology or the ability to reach the blastocyst stage (Table 3).

d) *4-HNE-protein conjugates assay*
  The FF of the largest follicles in the nF group contained a significantly higher level (6.8 times; *p* < 0.001) of 4-HNE-protein conjugates than those of F group (Fig. 3; panels a and b).
  The concentration of 4-HNE-protein conjugates did not affect day 2 embryo morphology or the ability to reach the blastocyst stage (data not shown).

### Expression of AMH and AMH receptor II genes in cumulus cells

**Fig. 4:**
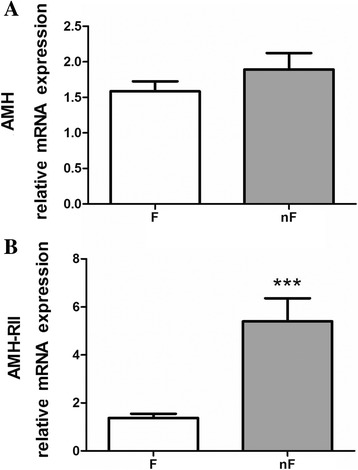
Expression of AMH and AMH receptor II genes in cumulus cells. Levels of messenger RNA (mRNA), expressed as relative fold change, of *AMH* (**a**) and *AMHRII* (**b**) genes in cumulus cells analyzed by quantitative real-time polymerase chain reaction (RT-qPCR, *n* = 3). F group = patients whose oocyte was fertilized (*n* = 29), nF group = patients whose oocyte was not (*n* = 17). *** *p* < 0.001

The mRNA expression of *AMH* gene was comparable in cumulus cells belonging to follicles whose oocyte was fertilized (F group) or not (nF group) (Fig. 4a), whereas *AMH-receptor II* gene mRNA expression was significantly higher (*p* < 0.02) in the cumulus cells of nF group (Fig. 4b). Noticeably, the mRNA expression of *AMH-receptor II* gene was significantly higher (*p* < 0.05) in cumulus cells coming from follicles with a high level of LDH (≥ 0.35 pmol NADH/min/mg proteins), whereas *AMH* gene was not influenced by FF-LDH level (data not shown).

### Multivariate analysis for the prediction of fertilization competence

Univariate analysis showed that LDH was strongly associated with fertilization (Odds ratio (OR): 1.5·10^−14^, 95%CI: 4.8·10^−14^-5·10^−4^), perfectly predicting the outcome. MS area and MS major axis were strongly collinear. Therefore, we fitted a multivariable logistic model for fertilization including age, FF-AMH, OSI, IL-ZP area, and MS area. ORs and 95% CIs are shown in Table 4. Age and OSI were associated with higher likelihood of fertilization (ORs 1.39 and 1.71, respectively), whereas FF-AMH and MS area with a lower probability (ORs 0.22 and 0.93, respectively). IL-ZP area was not associated with fertilization (OR 1.00).

**Table 4:** Association between the probability of fertilization (Odds Ratio (OR) with 95% confidence intervals (CI) and *p*-value) and selected variables

	Odd ratio	95%CI	*p*
Age	1.39	1.02–1.89	*p* < 0.05
FF-AMH	0.22	0.05–0.85	*p* < 0.05
OSI	1.71	1.11–2.63	*p* < 0.05
IL-ZP area	1.00	1.00–1.00	ns
MS area	0.93	0.86–1.00	*p* < 0.05

## Discussion

Identifying the most competent oocytes among those obtained during COS is still an important issue at least for two reasons: (a) it may help to understand which is the COS protocol able to obtain the best oocytes for each category of patients, allowing COS optimization and tailoring, and (b) it may avoid embryo overproduction with the consequent need of massive embryo cryostorage, which raises ethical problems and is still discouraged by law in some Countries (e.g. Italy). It is commonly accepted that the oocyte competence plays a central role in affecting the likelihood of fertilization and subsequent embryo development, even when fertilization is accomplished by direct injection of the sperm in the egg cytoplasm; as a consequence, selecting of the best oocytes would likely lead to an overall improvement of the efficacy of ICSI and of the average quality of in vitro produced embryos.

Oocyte morphological evaluation by stereomicroscopy and phase contrast microscopy is currently used for egg selection, but its accuracy is limited to the assessment of meiotic nuclear maturity [1]. Novel morphological approaches, such as polarized light microscopy (PLM), were proposed and tested in the last years, but still their results have not been fully convincing. Meiotic spindle detection by PLM during ICSI was claimed to predict fertilization [30], and MS morphology resulted to affect the likelihood of fertilization [4], embryo development [7] and conception [10]. Still, however, the data produced using PLM were not conclusive and for this reason the technique is not widely used.

Studying the oocytes derived from the largest follicle of each patient and comparing oocytes that were fertilized (F group) with those that were not (nF group), we observed that both the area and the length of the MS major axis were significantly higher in case of fertilization failure. This observation is different from what reported by Shen et al., 2005 [9], who observed that not MS size, but MS retardance, expressing the extent of molecular organization of the microtubules, was positively related to fertilization and embryo pronuclear score. Interestingly, we found bigger size MS more frequently among women of advanced reproductive age and lower ovarian sensitivity to exogenous FSH, suggesting that the increased MS size could represent a marker of oocyte ageing, in turn associated to maturation defects causing fertilization failure or meiotic errors. Interestingly enough, we also observed that the increased MS size was associated to an early embryo development arrest, before reaching the blastocyst stage. Indeed, the effect of oocyte aging on MS dynamics was well recognised as microtubule disorganization and altered morphology, often associated with fertilization failure or aneuploidy [31, 32]. This observation, however, suggests that any cause altering the maturation of egg cytoplasm could be reflected in some morphological changes of MS structure.

Since the acquirement of the competence toward fertilization and embryo development is influenced by a complex interplay between oocyte, CCs and FF, we applied a combined approach including oocyte morphology, the study of specific metabolites in FF and the expression of specific genes in CCs. We focused on those markers that were previously suggested to be implicated in the modulation of follicular growth and oocyte maturation, and we studied individual follicles in order to better assess the relationship between the follicle and the fate of the oocyte contained inside it.

The infra-follicular levels of AMH, whose serum concentration is a reliable marker of ovarian reserve [33] and responsiveness to COS [34], were reported by some authors to positively correlate with oocyte fertilization [12, 13]. This finding, however, was not confirmed in other studies [35–39], leaving several doubts about the role of FF-AMH as marker of oocyte competence. Herein we observed that FF-AMH was not correlated to oocyte fertilization competence, but was positively related to serum AMH, from which probably a relevant part of FF-AMH derives. We hypothesize that some previous positive reports about the role of FF-AMH as a marker of fertilization potential could have been biased by the fact that women with high serum AMH (and therefore high FF-AMH) are often young and consequently likely to produce competent oocytes.

The intra-follicular balance between oxidative stress and antioxidant systems is another issue that was shown to play a role during oocyte maturation, and to affect IVF outcome [40, 41]. Unfortunately most studies addressing the relationship between oocyte competence and FF redox status were performed on pooled follicles, and finally reached inconsistent conclusions [14–18] Tracking individual follicles and corresponding oocytes, we observed that the activity of Cu,Zn-SOD and catalase, two enzymes that neutralize reactive oxygen species [14], was significantly increased in the FF corresponding to nF oocytes, suggesting an increased need for antioxidant protection due to a higher level of oxidative damage in the FF of oocytes with poor fertilization potential. Our finding that the level of 4-hydroxynonenal-protein conjugates was significantly higher in the FF of nF oocytes confirms that oxidative stress, and lipo-peroxidation in particular [42], acts as an oocyte damaging condition, predisposing to fertilization failure. Noticeably, these observations agree with a previous study in which we observed that in cumulus cells the mRNA and protein expression of inducible nitric oxide synthase, heme-oxygenase-1 and IkBα (inhibitor of the redox-sensitive transcription factor NF-kB) phosphorylation - all pathways controlling redox-sensitive genes - was significantly related to fertilization failure [43].

Testing the FF concentration of LDH, a marker of cytotoxicity released outside when cell membrane is damaged [44], we observed that it was significantly higher in case of fertilization failure. Since the level of LDH in FF reflects cytotoxicity in the corresponding CCs, this finding suggests that a higher cytotoxic damage of CCs, with consequent leakage of LDH into the FF, may be associated with a loss of fertilization competence by the corresponding oocyte. FF-LDH concentration, however, was not related to the morphological variables observed using PLM, suggesting that the impairment of fertilization competence due to cytotoxicity could be determined by subtle abnormalities rather than to the induction of morphologically detectable defects of the oocyte.

Interestingly, the expression of *AMH-receptor II* gene in the CCs was significantly higher in follicles with elevated intra-follicular LDH. *AMH-receptor II* gene, furthermore, was significantly more expressed by CCs corresponding to nF oocytes than by those whose oocyte was normally fertilized.

Taken together, these observations suggest that an excessive cytotoxicity level, reflected in a higher activation of antioxidant defense mechanisms within the follicle, and an altered AMH signal transduction within CCs are strongly linked to fertilization outcome.

As AMH induces the redox-sensitive transcription factor NF-kB binding activity in breast and prostate cancer cells [45], it is possible that an induction of NF-kB in CCs, significantly related to fertilization failure [43], could be linked to the increase of AMH signal transduction in nF oocytes, in which the mRNA expression of *AMH-receptor II* gene and the oxidative status are greater than in normally fertilized oocytes.

All these factors impairing the oocyte fertilization competence could finally influence embryo development.

## Conclusions

In this study we identified a significant and independent association between fertilization failure after ICSI and specific biochemical, morphological and genetic oocyte-related parameters. The data presented herein could represent the basis of further studies aimed at assessing if some of these parameters could be prospectively used in the clinical practice as predictors of fertilization failure and inadequate oocyte competence.

## Additional file

Additional file 1:
**Figure S1.** Scheme of how material from individual first/largest follicle was evaluated in the enrolled patients’ population.

## Abbreviations

AFC: Antral follicle count
AMH: Anti-Müllerian Hormone
BMI: Body mass index
BSA: Bovine serum albumin
CCs: Cumulus cells
COC: Cumulus-oocyte complex
COS: Controlled ovarian stimulation
E2: Estradiol
ECL: Enhanced chemiluminescence
FF: Follicular fluid
FSH: Follicle-stimulating hormone
GnRH: Gonadotropin-releasing hormone
HCG: Human chorionic gonadotropin
HNE: 4-hydroxynonenal
ICSI: Intra-cytoplasmic sperm injection
IVF: In vitro fertilization
LDH: Lactate dehydrogenase
MS: Meiotic spindle
OPU: Ultrasound-guided oocyte retrieval
OSI: Ovarian sensitivity index
PLM: Polarized light microscopy
SOD: Cu,Zn-superoxide dismutase
ZP: Zona pellucida
