# Supplementary figures and images for: Oocyte polarized light microscopy, assay of specific follicular fluid metabolites, and gene expression in cumulus cells as different approaches to predict fertilization efficiency after ICSI
Source: Reprod Biol Endocrinol. 2017 Jun 23;15:47. doi: 10.1186/s12958-017-0265-2 (PMC5481970; doi:10.1186/s12958-017-0265-2)

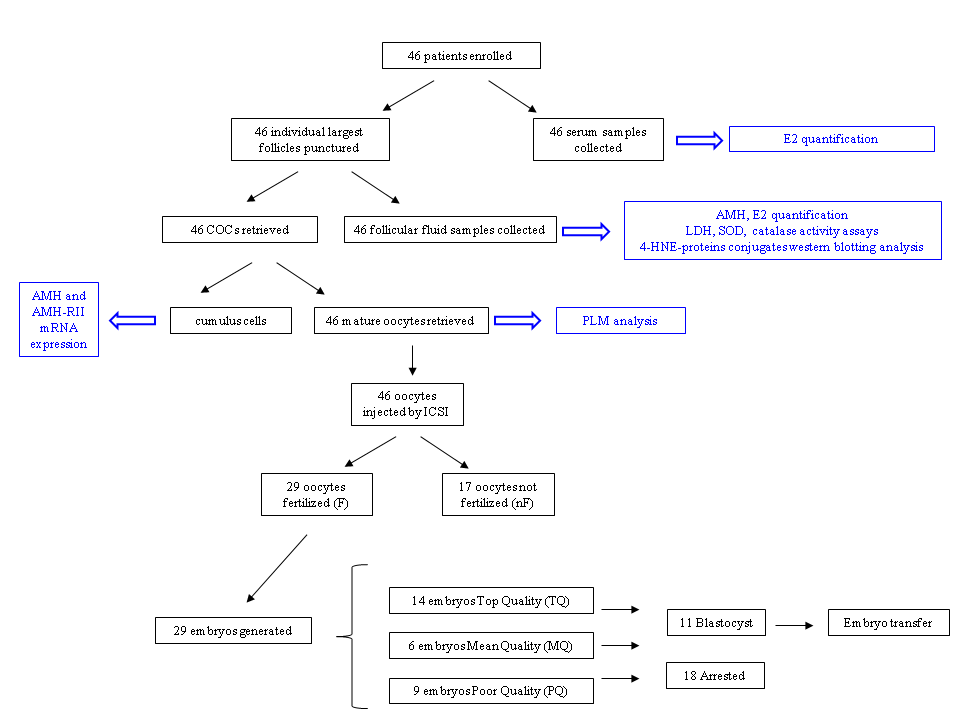

Supplement: Additional file 1: — Figure S1. Scheme of how material from individual first/largest follicle was evaluated in the enrolled patients’ population. [file 12958_2017_265_MOESM1_ESM.tif]
